# Hyperpolarization of ^15^N-pyridinium and ^15^N-aniline derivatives by using parahydrogen: new opportunities to store nuclear spin polarization in aqueous media†
†Electronic supplementary information (ESI) available: Synthetic procedures & characterization, NMR experimental description, computation of ^1^H and ^15^N enhancements. See DOI: 10.1039/c9sc02970b


**DOI:** 10.1039/c9sc02970b

**Published:** 2019-08-06

**Authors:** Anil P. Jagtap, Lukas Kaltschnee, Stefan Glöggler

**Affiliations:** a Max-Planck-Institute for Biophysical Chemistry , Am Fassberg 11 , 37077 Göttingen , Germany . Email: Stefan.gloeggler@mpibpc.mpg.de; b Center for Biostructural Imaging of Neurodegeneration , Von-Siebold-Str. 3a , 37075 Göttingen , Germany

## Abstract

Hyperpolarization techniques hold the promise to improve the sensitivity of magnetic resonance imaging (MRI) contrast agents by over 10 000-fold. Among these techniques, para-hydrogen induced polarization (PHIP) allows for generating contrast agents within seconds. Typical hyperpolarized contrast agents are traceable for 2–3 minutes only, thus prolonging tracking-times holds great importance for the development of new ways to diagnose and monitor diseases. Here, we report on the design of perdeuterated ^15^N-containing molecules with longitudinal relaxation times (*T*_1_) of several minutes. *T*_1_ is a measure for how long hyperpolarization can be stored. In particular, we introduce two new hyperpolarizable families of compounds that we signal enhanced with para-hydrogen: *tert*-amine aniline derivatives and a quaternary pyridinium compound with ^15^N-*T*_1_ of about 8 minutes. Especially the latter compound has great potential for applicability since we achieved ^15^N-polarization up to 8% and the pyridinium motif is contained in a variety of drug molecules and is also used in drug delivery systems.

## Abstract 2

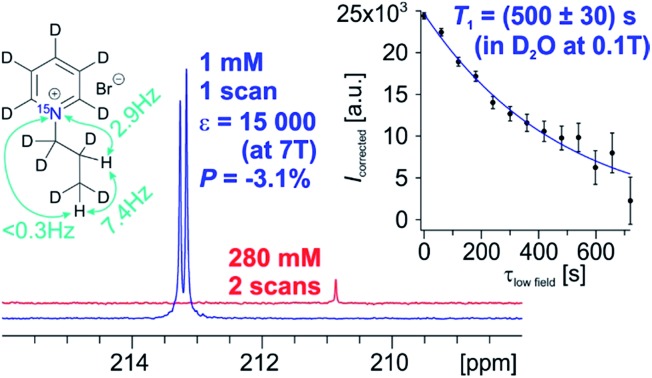
We introduce ^15^N quaternary pyridinium as moiety that can be NMR-signal-enhanced by several orders of magnitudes and allows for long-term storage of the so gained hyperpolarization in water.

## Introduction

Nuclear magnetic resonance (NMR) spectroscopy and magnetic resonance imaging (MRI) are powerful techniques, which have widely been used for studying molecular structures associated to diseases and to visualize illnesses even *in vivo*.^1^–^4^ Both techniques are greatly hampered, due to their low sensitivity. This limitation can be overcome by using hyperpolarization methods, which increase signals of molecules by more than four orders of magnitude.^5^–^13^ Several hyperpolarization techniques have evolved to gain new insights *e.g.* in the fields of structural biology, material science, chemical analysis, biochemistry and biomedical science. With a view on the latter, hyperpolarization allows for creating new contrast agents to study and diagnose diseases *in vivo*.^14^ The technique mainly used for producing hyperpolarized contrast agents is dissolution dynamic nuclear polarization (d-DNP).^5^ It enables the hyperpolarization of metabolically active compounds that can be followed during *in vivo* studies.^8^,^9^,^11^,^14^–^16^ Other methods with biomedical relevance are spin exchange optical pumping (SEOP)^17^–^20^ of noble gases and para-hydrogen induced polarization (PHIP).^21^–^32^ PHIP methods transfer nuclear spin order from para-hydrogen (para-H_2_) enriched hydrogen over to target molecules for their hyperpolarization. Hydrogenative PHIP adds para-H_2_ to unsaturated precursors over suitable hydrogenation catalysts, to create large spin-order in the target compounds, which can be converted into observable magnetization afterwards. Due to the design of suitable precursor molecules, this technique can now be utilized to hyperpolarize metabolically active compounds and to analyze their chemical conversion *in vivo*.^28^,^31^,^32^

Within the past ten years, a non-hydrogenative para-H_2_-based hyperpolarization methods has evolved: signal amplification by reversible exchange (SABRE).^32^–^37^ For this method, para-H_2_ and a substrate of interest coordinate to a temporarily stable transition metal complex. In this complex, the para-H_2_ spin order is converted into observable magnetization at the molecule of interest. Dissociation of the complex leads to free hyperpolarized substrates that have not been altered as in the classical PHIP approach.^38^ However, this method has not been shown to be applicable for *in vivo* applications yet since, SABRE experiments typically need to be performed in organic solvents. However, the field rapidly progressing and work is on the way to make this technique more biologically applicable in the future.^32^,^39^,^40^

What all techniques have in common is the desire to store hyperpolarization in contrast agents for long periods of time. To this end, hyperpolarization is typically stored on hetero-nuclei such as in ^13^C and ^15^N, which possess longitudinal relaxation times (*T*_1_) ranging from seconds to minutes. The *T*_1_ of ^13^C-pyruvate, the metabolite most commonly hyperpolarized, for example is in the range of 40–60 s.^41^ For *in vivo* applications, this results in a time window of 2–3 minutes, during which pyruvate can be monitored.^11^,^14^,^41^ To increase tracing times, ^15^N nuclei are more favorable than ^13^C nuclei since *T*_1_ can be one order of magnitude longer and *T*_1_ > 1200 s (20 minutes) in water have been reported in quaternary nitrogen compounds.^30^ Due to its longer *T*_1_ values, ^15^N-derived chemical probes have been explored: with respect to PHIP *N*-ethyl trimethyl ammonium (NETMA) and an allyl choline derivative have been polarized in biocompatible solvents.^30^,^42^,^43^ Dissolution DNP has demonstrated first *in vivo* experiments utilizing ^15^N polarized choline and several other applications *in vitro* such as pH-sensing, Ca^2+^ monitoring and enzyme activity.^44^–^46^ Degrees of ^15^N-polarization have long been rather low until the advancements in cross-polarization (CP) d-DNP have overcome this challenge.^13^

SABRE has made great progress in polarizing ^15^N spins in the past years.^34^,^35^,^47^ Demonstrations of over 40% polarization in ^15^N pyridine and more than 30% for imidazole have been accomplished in methanol.^48^,^49^ Prospective applications may include pH-sensing^50^ or probing of hypoxia.^47^,^49^ The later may in particular become feasible *via* storage of polarization in a ^15^N-nitro group of metronidazole which has a *T*_1_ of about 10 minutes in methanol.^51^

Currently the main challenge is to discover molecules that are biological relevant, have long *T*_1_ and can be hyperpolarized to a large degree. Here, we are tackling this challenge and introduce classes of compounds that meet these requirements. Our particular focus is thereby on pyridinium, a compound already relevant in drug applications.^52^–^56^

## Experimental

**Scheme 1:**
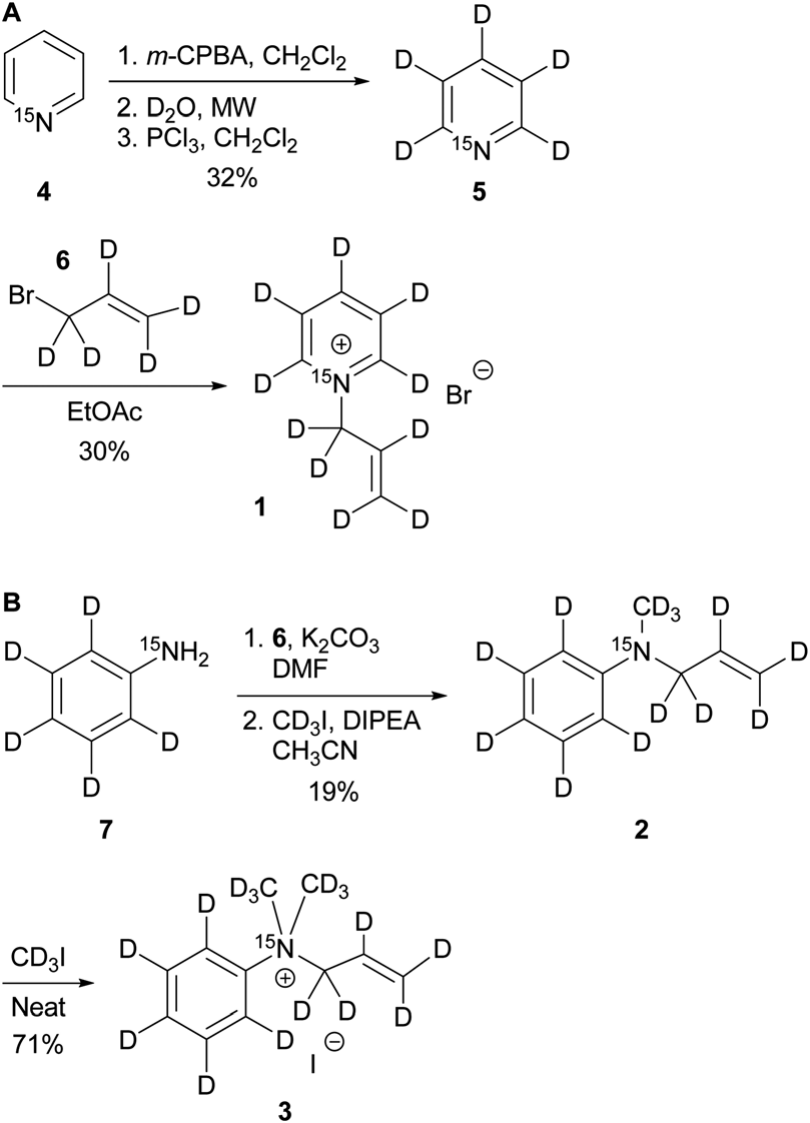
Syntheses of ^15^N-pyridinium derivative (A) and -aniline based (B) derivatives; MW: microwave.

The synthesis of the labelled compounds was conducted as follows: to yield **1**, we prepared ^15^N-pyridine-*d*_5_ starting from protonated ^15^N-pyridine, oxidation with *meta*-chloroperoxybenzoic acid (*m*-CPBA) followed by H–D exchange reaction under microwave condition in D_2_O (Scheme 1A). Further reduction with PCl_3_ in CH_2_Cl_2_ yielded ^15^N-pyridine-*d*_5_.^57^ Finally, quaternization of **5** was accomplished by the treatment with allyl bromide-*d*_5_ (**6**) in EtOAc to yield **1** as colourless solid.^58^ In order to synthesize the aniline derivatives **2** and **3**, we first synthesized ^15^N-aniline-*d*_5_ (**7**) in a two-step procedure from benzene-*d*_5_.^59^ Mono-allylation of **7** with allyl bromide-*d*_5_ (**6**) in the presence of K_2_CO_3_ and further treatment with CD_3_I in presence of DIPEA, yielded **2** (Scheme 1B). Stirring of **2** in neat CD_3_I leads to the quaternary aniline derivative **3**. Further experimental details can be found in the ESI.†

## Result and discussion

We have synthesized and investigated a library of ^15^N-enriched compounds and report on two novelties: firstly, we have discovered an aniline derivative containing a tertiary amine with a long *T*_1_ of about 10 minutes in methanol-*d*_4_ (MeOD). This is of particular interest since it demonstrates that uncharged nitrogen species, in addition to quaternary compounds, have potential to store polarization for long periods and opens up new possibilities to design contrast agents with lipophilic moieties. Secondly, we are introducing a new class of compounds that can be hyperpolarized and possesses a *T*_1_ of about 8 minutes in water: quaternary pyridinium derivatives. Quaternary pyridinium is a core structure found in many molecules which has been used for investigations of neurodegenerative diseases^60^ as well as in drug design and drug-delivery approaches.^52^–^56^ We furthermore present the hyperpolarization of the library of compounds *via* PHIP and a pulsed transfer method to enhance the ^15^N signals. Generating contrast agents in aqueous media becomes possible by utilizing rhodium nano-catalysts (NAC@Rh) that promote the hydrogenation reaction with para-H_2_ in water.^30^

Table 1 presents the investigated compounds and at the top the general scheme of how the investigated compounds are hyperpolarized with para-H_2_. The precursor compounds prior to hydrogenation are a pyridinium derivative (**1**), a *tert*-amine derivative of aniline (**2**) and a quaternary nitrogen derivative of aniline (**3**). We have perdeuterated all of the precursors to prolong ^15^N-*T*_1_ by weakening dipolar couplings, as compared to the protonated counterparts. As an unsaturated moiety to which para-H_2_ will be added during the hydrogenation step, we have chosen deuterated allyl groups. The rationale behind this choice is twofold: first, the added protons from para-H_2_ after the hydrogenation will be one extra bond away as compared to the vinyl derivatives, thus reducing dipolar interactions that potentially shorten *T*_1_. Second, the scalar coupling network in the hydrogenation products **1a–3a** are thought to be an ideal spin system to apply the recently developed ESOTHERIC (efficient spin order transfer to heteronuclei *via* relayed INEPT chains) spin order transfer sequence to hyperpolarize the ^15^N spins.^61^,^62^ This is because the ^3^*J*_H,N_ coupling is larger than ^4^*J*_H,N_ (see ESI†) and the protons are weakly coupled.

**Table 1:** ^15^N-*T*_1_ values for pyridinium and phenylammonium compounds along with their reduced products using para-H_2_^*a*^

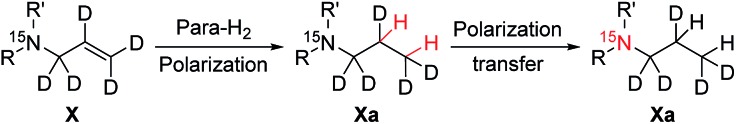				
X	Structure	^15^N-*T*_1_ in s	Mag. field in T	Solvent and temp. in K
**1**	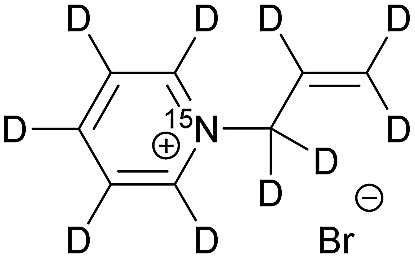	140 ± 20	7	MeOD at 320
		220 ± 30	7	D_2_O at 353
**1a**	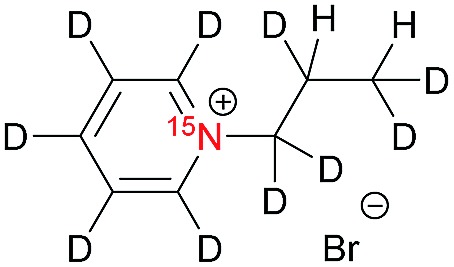	240 ± 10^*b*^	7	MeOD at 320
		360 ± 50^*b*^	1	MeOD at 320
		570 ± 110^*b*^	0.1	MeOD at 320
		380 ± 30^*b*^	0.01	MeOD at 320
		120 ± 10^*c*^	7	D_2_O at 353
		330 ± 20^*c*^	1	D_2_O at 353
		500 ± 30^*c*^	0.1	D_2_O at 353
		290 ± 20^*c*^	0.01	D_2_O at 353
**2**	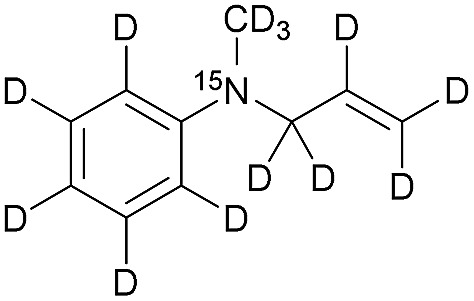	570 ± 40	9.4	MeOD at 298
**2a**	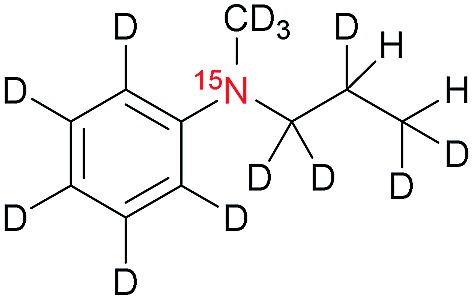	150 ± 20^*c*^	7	MeOD : D_2_O at 320
		90 ± 40^*c*^	0.1	MeOD : D_2_O at 320
**3**	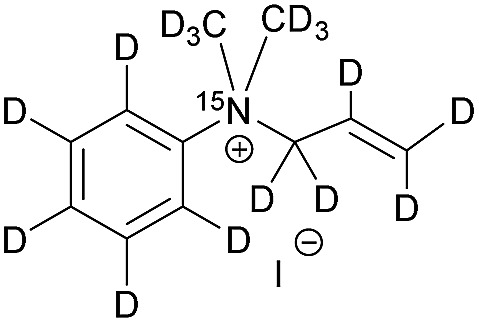	420 ± 100	9.4	D_2_O at 298
**3a**	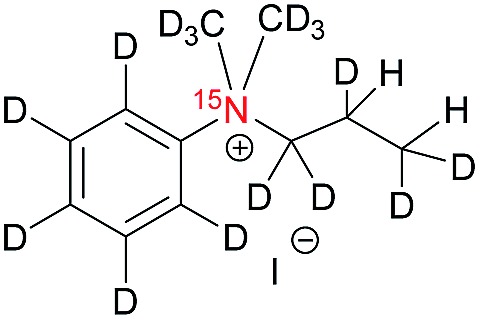	Decomposed	—	—

^*a*^A general scheme of hyperpolarization followed by polarization transfer to ^15^N nuclei; X = **1** (R, R′ = pyridinium); X = **2** (R = phenyl, R′ = –CD_3_) and X = **3** (R = phenyl, R′ = –(CD_3_)_2_); Mag.: magnetic; temp.: temperature.

^*b*^In the presence of 2 mM [Rh(dppb)(COD)][BF_4_].

^*c*^In the presence of 0.5 mg mL^–1^ NAC@Rh.

Prior to performing hyperpolarization experiments, we determined ^15^N-*T*_1_ for the unsaturated precursor molecules **1–3** in D_2_O, MeOD or mixtures thereof to increase the molecule's solubility. The ^15^N-*T*_1_ values obtained in different solvents and at various magnetic fields are summarized in Table 1. For the precursor molecules it is noteworthy to mention that ^15^N-*T*_1_ of the *tert*-amine **2** has a ^15^N-*T*_1_ of 570 ± 40 s in MeOD (this compound was not soluble in water) at high field and the quaternary ammonium compound **3** displays a ^15^N-*T*_1_ of 420 ± 100 s in D_2_O. For the unsaturated pyridinium derivative **1**, we discovered a ^15^N-*T*_1_ of 220 ± 30 s at high field in D_2_O.

Since we found ^15^N-*T*_1_ values of several minutes for all precursor compounds, we performed hydrogenation reactions and investigated ^15^N-*T*_1_ of the hydrogenation products. This was done by hyperpolarizing the ^15^N nuclei and measuring the polarization decay with low flip angle pulses as described in the next paragraph and in the ESI.† Our first observation was that the anilinium derivative **3a** decomposes upon hydrogenation. This may reflect that trimethylanilinium is typically used as a methylation agent^63^ and not stable enough for hyperpolarization studies with para-H_2_. Moreover, a similar kind of degradation was reported on ^15^N-propargylcholine while performing PHIP.^42^ In addition to this, Shchepin *et. al.* reported lack of the successful ^15^N hyperpolarization on other choline derivatives using ^15^N-enriched PHIP precursors.^64^ The ^15^N-*T*_1_ of the *tert*-amine **2a** is strongly reduced after hydrogenation to 150 ± 20 s. Lastly, the pyridinium derivative **1a** has a *T*_1_ of 120 ± 10 s at high field in D_2_O, but reaches 500 ± 30 s (about 8 minutes) when the field is lowered to 0.1 T (see also Fig. S1†). With respect to *T*_1_, the main relaxation source at high field appears to be chemical shift anisotropy (CSA). This offers possibilities to make the compound applicable for studies in clinical scanners. Given its long ^15^N-*T*_1_ at low field in water and being an important structure in a variety of biomolecules or drugs, the pyridinium derivative is the most promising compound discovered among the investigated compounds here for future applications.

To obtain the hyperpolarized products, compounds **1–3** were hydrogenated with para-H_2_ under two experimental conditions: for preparation in MeOD, we used the homogeneous Rh-catalyst [Rh(dppb)(COD)][BF_4_] (dppb: diphenylphosphino butane, COD: cyclooctadiene). For hyperpolarization in D_2_O, we used an *N*-acetylcysteine-capped Rh-nano-catalysts (NAC@Rh).^30^ The enrichment of H_2_ in its *para*-state was 80%, as determined experimentally. At first, we have investigated the ^1^H polarization and subsequently the ^15^N polarization following the ESOTHERIC sequence.^61^,^62^ The results are summarized in Table 2.

**Table 2:** PHIP enhancements by using the homogenous and heterogeneous catalysts

	Compd.	^1^H *P*%	^15^N *P*%
PHIP (homogeneous) MeOD at 320 K	**1a**	11 ± 1.3	7.4 ± 0.6
	**2a**	—^*a*^	—^*a*^
	**3a**	—^*a*^	—^*a*^
PHIP (heterogeneous) D_2_O at 353 K	**1a**	2.1 ± 1.2	2.3 ± 1.1
	**2a**	1.3 ± 0.2^*b*^	0.8 ± 0.1^*b*^
	**3a**	—^*a*^	—^*a*^

^*a*^Multiple products or decomposition.

^*b*^Measured in MeOD : D_2_O (1 : 1) at 320 K. Compd.: compound.

As compound **3a** did not form during hydrogenation, no hyperpolarization data is reported here for either the homogeneous or heterogeneous catalyst. Compound **2** turned out to be insoluble in D_2_O; therefore, we chose an equimolar mixture of MeOD and D_2_O for dissolving the heterogeneous catalyst for PHIP experiments. We have found 1% polarization of ^1^H and ^15^N nuclei respectively in the hydrogenated compound **2a**, whereas multiple polarized products were observed in MeOD with the homogeneous catalyst. This result demonstrates that heterogeneous catalysts provide new opportunities for polarizing nitrogen containing compounds that may not be accessible with the standard homogeneous catalyst.

**Fig. 1:**
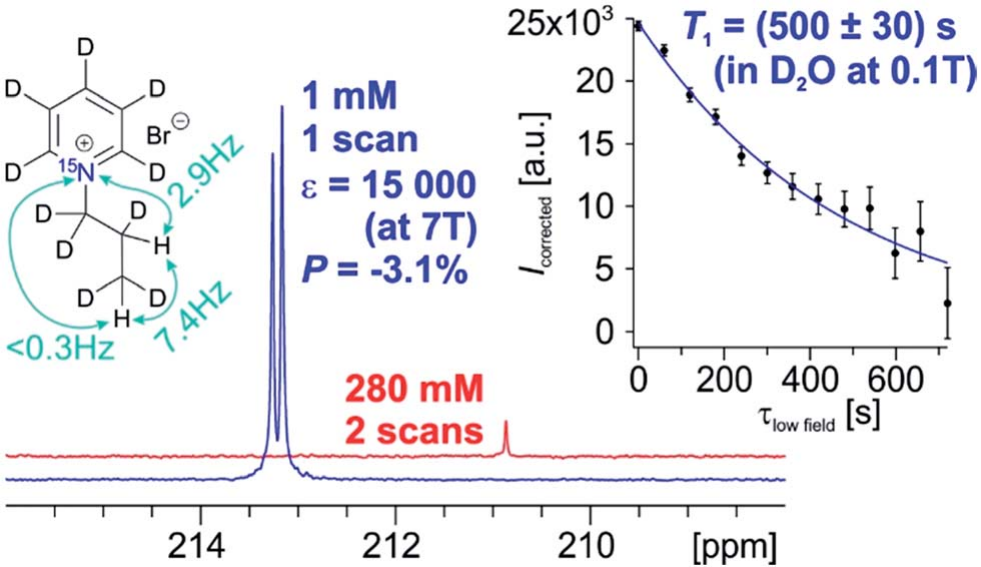
Hyperpolarized ^15^N spectrum of **1a** (blue) by using PHIP in D_2_O and thermally polarized ^15^N spectrum of the unsaturated precursor **1** (red) at 7 T. The inset shows the ^15^N-*T*_1_ relaxation data measured at 0.1 T, using a hyperpolarized sample of **1a** which was collected using sample shuttling and small flip angle pulses (see ESI† for further details).

With respect to the pyridinium derivative, we observed significant ^1^H polarization of 11% ± 1.3% in **1a** using the homogeneous catalyst in MeOD. We succeeded in transferring this polarization to the ^15^N-spin with a signal enhancement (*ε*) of 32 000 (*P* = 7.4% ± 0.6%) compared to thermal polarization at *B*_0_ = 7 T at 320 K in MeOD. For improved biocompatibility, we performed polarization experiments with the heterogeneous catalyst in water and achieved a highest polarization of 3.1% (*ε* = 15 000-fold compared to the thermal signal at 353 K, Fig. 1) and an average 2.3% polarization. The spectrum of the hyperpolarized compound in water as well as the *T*_1_-experiment (inset) with small tip angle pulses at 0.1 T is depicted in Fig. 1.

## Conclusions

In conclusion, we have introduced and synthesized perdeuterated ^15^N-allyl-pyridinium (**1**) and -aniline derivatives (**2** & **3**). We succeeded in forming hyperpolarized addition products of **1** and **2** utilizing para-H_2_. Most notably, a ^15^N-pyridinium derivative (**1a**) provided strong ^15^N-polarization of *P* = 7.4% in methanol and *P* = 2.3% in water compared to thermal polarization. Polarization in water was achieved *via* rhodium nanocatalysts that although heterogeneous PHIP catalysts are still in an early development stage show here the possibility to signal enhance molecules that are not polarizable with standard homogeneous metal complexes. In water at 0.1 T field, we discovered a long ^15^N-*T*_1_ of about 8 min. We also found that the *tert*-amine **2** features notably a slow relaxation time of 10 min for ^15^N-nuclei in methanol. This is despite the fact that it is not a quaternary nitrogen compound, and thus could be used as a hydrophobic ^15^N-labelled tracer. Overall, our presented studies introduce new possibilities for the molecular design of contrast agents and storage capabilities of hyperpolarized spin states. It is noteworthy to mention that out of all compounds studied here, the highest levels of hyperpolarization (^1^H and ^15^N) were found in pyridinium derivatives, a molecular species present in many bio-relevant molecules. Longer relaxation times of ^15^N nuclei of these compounds in combination with targeting moieties will potentially in the future ensure long traceability and opportunity to deliver the hyperpolarization in organisms for biomedical imaging applications.

## Conflicts of interest

There are no conflicts to declare.

## Supplementary Material

Supplementary information
