# Supplementary material for: Hyperpolarization of 15N-pyridinium and 15N-aniline derivatives by using parahydrogen: new opportunities to store nuclear spin polarization in aqueous media
Source: Chem Sci. 2019 Aug 6;10(37):8577–82. doi: 10.1039/c9sc02970b (PMC6839503; doi:10.1039/c9sc02970b)
Supplement: Supplementary file 1 [file SC-010-C9SC02970B-s001.pdf]

Electronic Supplementary Material (ESI) for Chemical Science.
This journal is © The Royal Society of Chemistry 2019

Hyperpolarization of ^{15}N-pyridinium and ^{15}N-aniline derivatives by using parahydrogen: New opportunities to store nuclear spin polarization in aqueous media

Anil P. Jagtap[a,b], Lukas Kaltschnee[a,b] and Stefan Glöggler*[a,b]

Table of contents

Abbreviations 3
Synthetic procedures 3
General materials and methods 3
Synthetic protocols 4
^{15}N-pyridinium derivative (**1**) 4
^{15}N-Compound (**2**) 5
^{15}N-phenylammonium derivative (**6**) 6
NMR Instrumentation used 7
^{15}N-T_1 measurements using inversion recovery (compounds **1**, **2** & **3**) 7
Parahydrogen-induced polarization (PHIP) experiments 7
Sample & nuclear spin order preparation 7
^{1}H hyperpolarization 8
^{15}N hyperpolarization 9
^{15}N-T_1 measurements for compounds **1a** & **2a** 9
^{15}N-T_1 field dependence for compound **1a** 11
Signal assignment and coupling values in the hydrogenation products **1a** and **2a** 12
References 12

Abbreviations

DIPEA	*N*,*N*-Diisopropylethylamine
EtOAc	Ethyl acetate
ESOTHERIC	efficient spin order transfer to heteronuclei via relayed INEPT chains
Et_3N	Triethylamine
ESI-MS	Electrospray ionization mass spectrometry
TLC	Thin layer chromatography

Synthetic procedures

General materials and methods

Chemicals were purchased from commercial suppliers and were used without further purification. Allyl-d_5 bromide (**6**) was purchased from CDN Isotopes Inc. (Product number: D-3987) . Compounds **5**[1], **7** (^{15}N-aniline d_5)[2] and **8**[3] were prepared analogously with the reported procedures. Thin layer chromatography (TLC) was performed on glass backed TLC plates and compounds were visualized by UV light. Silica gel (230-400 mesh, 60 Å) was purchased from Aldrich and used for flash chromatography. ^{1}H, ^{2}H, ^{13}C and ^{15}N NMR spectra were recorded on Bruker Avance III HD spectrometers, at 7 T or at 9.4 T. Further details on the instrumentation can be found below in the section "NMR Instrumentation used". The signals of the deuterated solvents were used as internal chemical shift standards. Residual proton signals from the deuterated solvents were used as references [D_2O (4.81 ppm), $CDCl_3$ (7.26 ppm), methanol-d_4 (3.31 ppm)] for ^{1}H spectra. The residual ^{13}C signals from the deuterated solvents being used as references [$CDCl_3$ (77.0 ppm), methanol-d_4 (49.05 ppm)] for ^{13}C spectra. All coupling constants were measured in Hertz. All moisture sensitive reactions were carried out in oven-dried glassware using nitrogen industrial grade cylinders. Molecular masses of the new organic compounds were determined by ESI-MS (Bruker).

Synthetic protocols

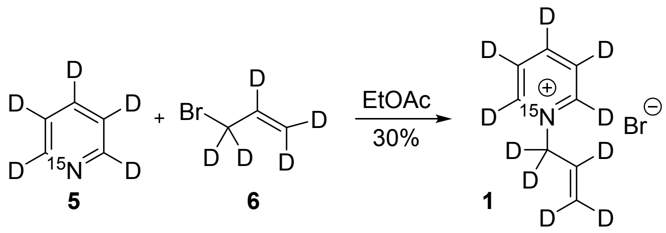

^{15}N-pyridinium derivative (1)

To a cold solution of ^{15}N-pyridine-d_5[1] (**5**, 0.093 g, 1.09 mmol) in 3 mL anhydrous ethyl acetate at 0 °C, allyl bromide-d_5 (**6**, 0.095 mL, 1.05 mmol) was added dropwise. The resulting reaction mixture was stirred for 48 h at 27 °C. The solid obtained was filtered out and washed with anhydrous ethyl acetate and anhydrous diethyl ether three times each to remove unreacted precursor material. The product was subsequently dried under reduced pressure to obtain **1** (0.035 g, 30% on recovery basis of the staring material) as white crystalline solid.

TLC (Silica gel, 20% MeOH in CH_2Cl_2), R_f (**5**) = 1, R_f (**1**) = 0.2, UV active.
(Silica gel, 20% EtOAc in pet. ether), R_f (**5**) = 0.2

^{2}H NMR (D_2O, 353 K, 46.1 *MHz*) δ = 9.38 (2 x ^{2}H), 9.16 (1 x ^{2}H), 8.68 (2 x ^{2}H), 6.73 (1 x ^{2}H), 6.13(1 x ^{2}H), 6.03 (1 x ^{2}H), 5.74 (2 x ^{2}H) ppm.

^{13}C NMR (D_2O, 353K, 75.5 *MHz*) = 146.3 (J_{15N} = 5.2 Hz), 144.5 (J_{15N} = 13 Hz), 130.1 (J_{15N} = 1.43 Hz), 128.6 (J_{15N} = 2 Hz), 123 (J_{15N} = 2 Hz), 63.2 (J_{15N} = 7 Hz) ppm.

^{15}N NMR (D_2O, 353K, 30.4 *MHz*) = 210.9 ppm.

HRMS: calculated for $C_8D_{10}{}^{15}N$: 131.1406, found 131.1410 (M-Br)$^+$.

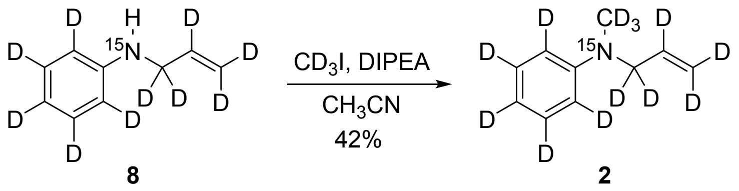

^{15}N-Compound (2)

To a solution of **8**[2-3] (0.18 g, 1.25 mmol) in CH_3CN (4 mL) was added DIPEA (1.4 mL, 8.79 mmol), CD_3I (0.470 mL, 7.53 mmol) and Et_3N (0.073 mL, 0.525 mmol) under an inert atmosphere of argon. The resulting solution was stirred for 24 h at 27 °C. After completion of the reaction, the solvent was removed *in vacuo*. The crude product was purified by flash column chromatography (silica) using a gradient elution (EtOAc: pet ether; 0:100 to 5:95) to give **8** (0.085 g, 42% yield) as a yellowish liquid.

TLC (Silica gel, 5% EtOAc in Pet. ether), R_f(**8**) = 0.3, R_f(**2**) = 0.4, UV active.

^{2}H NMR (MeOD, 298 K, 46.1 *MHz*) δ = 7.19 (2 x ^{2}H), 6.76 (2 X ^{2}H), 6.67 (1 x ^{2}H), 5.85 (1 x ^{2}H), 5.13(2 x ^{2}H), 3.86 (2 x ^{2}H), 2.85 (3 x ^{2}H) ppm.

^{13}C NMR (MeOD, 298K, 75.5 *MHz*) = 149.5 (J_{15N} = 13.7 Hz), 133.2 (J_{15N} = 0.4 Hz), 128.0 (J_{15N} = 1.2 Hz), 115.7 (J_{15N} = 0.4 Hz), 114.5 (J_{15N} = 1.7 Hz), 112.0 (J_{15N} = 2.1 Hz), 53.9 (J_{15N} = 10.4 Hz), 36.1 (J_{15N} = 10.8 Hz) ppm.

^{15}N NMR (MeOD, 298K, 40.54 *MHz*) = 50.7 ppm.

HRMS: calculated for $C_{10}D_{13}{}^{15}N$: 161.1907, found 162.1911 $(M+H)^+$.

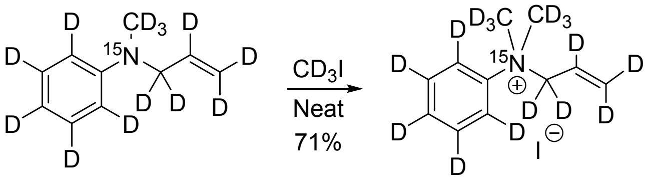

2 3

^{15}N-phenylammonium derivative (6)

The clear solution of **2** (0.060 g, 0.372 mmol) was stirred in CD_3I (0.6 mL) for 72 h at 27 °C. The solid obtained was filtered and washed with 5% EtOAc in Pet. Ether (3 x 3 mL) to yield **3** (0.080 g, 71% yield) as a white solid.

TLC (Silica gel, 5% EtOAc in pet. ether), R_f (**2**) = 0.4, R_f (**3**) = 0, UV active.

^{2}H NMR (MeOD, 298 K, 46.1 *MHz*) δ = 7.7 (3 x ^{2}H), 8.01 (2 x ^{2}H), 5.66 (3 x ^{2}H), 4.6 (2 x ^{2}H), 3.7(6 x ^{2}H) ppm.

^{13}C NMR (MeOD, 298K, 75.5 *MHz*) = 144.4 (J_{15N} = 1.2 Hz), 129.8 (J_{15N} = 0.4 Hz), 127.5 (J_{15N} = 1.9 Hz), 124.4 (J_{15N} = 0.9 Hz), 120.5 (J_{15N} = 1.2 Hz), 70.2 (J_{15N} = 3.7 Hz), 52.3 (J_{15N} = 5.5 Hz) ppm.

^{15}N NMR (MeOD, 298K, 40.54 *MHz*) = 63.5 ppm.

HRMS: calculated for $C_{11}D_{16}{}^{15}N$: 179.2252, found 179.2253 $(M\text{-}Br)^+$.

NMR Instrumentation used

Most experiments were performed on a Bruker Avance III HD NMR spectrometer with a 7 T ultrashield magnet (^{1}H: 300.13 MHz, ^{13}C: 75.47 MHz, ^{2}H: 46.07 MHz, ^{15}N: 30.41 MHz). The system was equipped with a room temperature 5 mm broadband direct observe double resonance probe (BBFO-H-D) equipped with a z-gradient. 90° hard pulses used with this probe were 9.5µs for ^{1}H, 14 µs for ^{13}C, 15 µs for ^{15}N and 200 µs for ^{2}H. A BCU I using was used for temperature regulation. For para-hydrogen experiments, magnetic valves controlling the pressurization and bubbling of the sample are controlled by the spectrometers TTL output signals. For pressurized samples, a backpressure valve constantly keeps the sample pressure at 7 bar. A Bruker para-hydrogen generator (BPHG 90) was used to deliver para-H_2 enriched hydrogen at pressures above 7 bar. The para-H_2 enrichment was experimentally found to be around 80%, as determined by comparing the ^{1}H signal integral of enriched gaseous hydrogen with that of hydrogen that was not enriched, at the same gas pressure (7 bar).

Further experiments were performed on a Bruker Avance III HD spectrometer operating a 9.4 T shielded magnet (^{1}H: 400.13 MHz, ^{13}C: 100.61 MHz, ^{2}H: 61.42 MHz, ^{15}N: 40.54 MHz). The system was equipped with a room temperature 5 mm inverse quadruple resonance probe (QXI-H/P-C/N-D) equipped with a z-gradient. 90° hard pulses used with this probe were 9.75µs for ^{1}H and 42.5 µs for ^{15}N. A BCU II using was used for temperature regulation.

^{15}N-T_1 measurements using inversion recovery (compounds 1, 2 & 3)

^{15}N-T_1 values for compounds **1** were measured at 7 T with an inversion recovery experiment with ^{2}H decoupling during acquisition. Solutions of **1** (280 mM) in either methanol-d_4 or D_2O were prepared in Young tubes and degassed via three freeze-pump-thaw cycles. ^{15}N pulses (16 µs for 90°) were centred on the ^{15}N resonance of **1**. Spectra were collected with two scans and a recovery delay of 30 min. The fit was performed with six timepoints (τ = 7s, 20s, 60s, 300s, 900s, 1800s).

^{15}N-T_1 values for compounds **2** and **3** were measured at 9.4 T with an inversion recovery experiment without ^{2}H decoupling during acquisition. Solutions of **2** (350 mM) and of **3** (200 mM) were prepared in methanol-d_4 in Young tubes and degassed via three freeze-pump-thaw cycles. ^{15}N pulses (9.75 µs for 90°) were centred at the ^{15}N resonance frequencies. Spectra were collected with two scans and a recovery delay of 60 min. The fit was performed with five timepoints (τ = 10s, 60s, 180s, 900s, 3600s).

Parahydrogen-induced polarization (PHIP) experiments

Sample & nuclear spin order preparation

Hyperpolarization experiments with para-H_2 were performed using the 7 T system, described above. Experiments with the homogeneous catalyst [Rh(dppb)(COD)][BF_4] were performed in in methanol-d_4 with 2 mM substrate and 4 mM catalyst concentration. Experiments with the heterogeneous catalyst NAC@Rh were performed with 1 mM substrate and 0.5 mg/mL

catalyst concentration. For compounds **1** & **3**, D_2O was used as solvent, whereas 1:1 D_2O/methanol-d_4 was used for **2** to increase solubility. N_2 was supplied to the samples for oxygen removal, then pressurized with N_2 at 7 bar and inserted into the magnet.

After temperature equilibration, 7 bar para-H_2 pressure (80% enrichment in para-H_2) were applied and the samples were suppliedwith para-H_2 for variable times (see Table S1) followed by a 1s delay for sample homogenization.

Table S1: Summary of experimental settings used for ^1H and ^{15}N hyperpolarization experiments.

	catalyst	bubbling duration [s]	c_0 [mM]	T [K]	solvent
1a	[Rh(dppb)(COD)][BF_4]	30	2	320	MeOD
2a	[Rh(dppb)(COD)][BF_4]	10	2	320	MeOD
3a	[Rh(dppb)(COD)][BF_4]	20	2	320	MeOD
1a	NAC@Rh	13	1	353	D_2O
2a	NAC@Rh	30	1	320	1:1 D_2O/MeOD
3a	NAC@Rh	20	1	353	D_2O

^{1}H hyperpolarization

For ^{1}H hyperpolarization experiments, a hard 45° ^{1}H pulse was applied, and the FID was detected directly after. The signal integral (magnitude) I_{PHIP} was then compared to the signal integrals measured in reference spectra, previously acquired for computation of the signal enhancement factors (see below). Enhancement factors ε were computed according to

$$\varepsilon = \frac{I_{PHIP}}{I_{m,ref} c_0}, \quad \text{(S1)}$$

where $I_{m,ref}$ is the reference integral expected for one mole of thermally polarized nuclei at the given field and temperature, and c_0 is the initial concentration of the unsaturated precursor.

Polarizations P were then computed, according to

$$P = q\varepsilon P_{thermal}, \text{ with } P_{thermal} = \frac{e^{-\frac{1}{2}K} - e^{+\frac{1}{2}K}}{e^{-\frac{1}{2}K} + e^{+\frac{1}{2}K}} \text{ and } K = \frac{\gamma \hbar B_0}{k_B T}, \quad \text{(S2)}$$

where $P_{thermal}$ is the thermal polarization expected for an ensemble of identical spin-1/2 nuclei at the given field B_0 and temperature T, $q = 2$ is a factor accounting to the fact, that the 45° pulse used converts only half of the polarization created into observable anti-phase signals[4], γ is the gyromagnetic ratio of the nucleus, $\hbar$ is the Planck constant divided by 2π, B_0 is the static magnetic field and k_B is the Boltzmann constant.

The reference spectra had been acquired under conditions ensuring full thermal relaxation (relaxation delay > 5 T_1) and using 90° pulses, but with otherwise identical instrument settings. The four reference samples used were 500 mM maleic acid in D_2O, 400 mM succinic acid in D_2O, 50 mM maleic acid in methanol-d_4 and 50 mM diethyl malonate in methanol-d_4. For a

given temperature, the reference molar integrals agreed within a margin of ± 4%, with no apparent influence of the solvent. The above-mentioned reference integrals $I_{m,ref}$ were computed according to

$$I_{m,ref} = \frac{I_{ref}}{c_{ref}\eta_{ref}NS}, \qquad \text{(S3)}$$

where $I_{ref,k}$ is the integral used for referencing, c_{ref} is the reference material concentration, η_k is the number of chemically equivalent nuclei contributing to the reference signal and NS is the number of scans acquired.

^{15}N hyperpolarization

For ^{15}N hyperpolarization experiments, the ESOTHERIC[5] sequence in Fig. 1 2) of ref. [5b] was used without the final ^{15}N 90° pulse (and using ^{15}N instead of ^{13}C as the X-nucleus). For compound **1a**, the delays Δ1 = 189 ms and Δ2 = 68.4 ms were used, for the heteronuclear and for the homonuclear coupling evolution periods, respectively. For compound **2a**, the delays Δ1 = 306 ms and Δ2 = 67.6 ms were used. For compound **3a**, various settings for these delays were used, but no hyperpolarization of the ^{15}N nucleus could be achieved.

^{15}N polarizations were computed according to equations (*S1*) - (*S3*), in full analogy to ^{1}H polarizations. ^{15}N reference spectra were collected for 100 mM urea[$^{15}N_2$] sample prepared in 90% H_2O/10% D_2O, as well as for compound **2**, 280 mM in D_2O or in methanol-d_4. Full relaxation (relaxation delay > 5 T_1) was ensured. For a given temperature, the reference molar integrals agreed within a margin of ± 3%, with no apparent influence of the solvent.

^{15}N-T_1 measurements for compounds 1a & 2a

^{15}N-T_1 for the reduced compounds **1a** & **2a** were measured on ^{15}N-hyperpolarized samples. Identical conditions were used, as for the ^{15}N-hyperpolarization experiments, with the exception, that the final ^{15}N 90° pulse was used, to flip the polarization generated to the z-axis. To measure the ^{15}N-T_1, the polarization step was followed by a series of ^{15}N spectra, acquired with a small excitation angle θ. ^{15}N pulses of 0.5 μs (3°) were used for **1a** in methanol-d_4, of 3 μs (18°) were used for **1a** in D_2O and of 1 μs (6°) were used for **2a**.

To measure the ^{15}N-T_1 at 7 T, the samples resided inside the probe during the measurement, and fixed repetition delays of 60 s (**1a**) or 30 s (**2a**) were used. Prior to fitting, the signal integrals obtained at these high field measurements were corrected for magnetization loss caused by preceding pulses with tilt angle θ, according to

$$I_{corrected,HF}(n) = \frac{I_0(n)}{(\cos\theta)^{(n-1)}}, \qquad \text{(S4)}$$

where $I_0(n)$ is the signal integral extracted from the nth spectrum in the small flip angle series, and $I_{corrected,HF}(n)$ in the corrected signal integral. These corrected integrals were fit according to

$$I_{corrected,HF}(n) = M_0 e^{-\frac{t(n)}{T_{1,HF}}} , \qquad (S5)$$

to extract the high field T_1 value $T_{1,HF}$.

To avoid a bias of the fit through this upscaling procedure of late time points, the fits were performed with non-uniform weighing of the data points. To this end, the signal-to-noise ratio $\left(S/N\right)_{n=1}$ was determined in the first scan of the data series, and it was assumed, that

$$\Delta I_{corrected,HF}(n) = \frac{I_0(n=1)}{\left(S/N\right)_{n=1}} \frac{1}{(\cos\theta)^{(n-1)}} , \qquad (S6)$$

provides a reasonable error estimate for $I_{corrected,HF}(n)$. During the fitting, all data points were weighed, according to

$$w_{HF}(n) \propto \left(\Delta I_{corrected,HF}(n)\right)^{-2} . \qquad (S7)$$

To measure the ^{15}N-T_1 at lower fields, the B_0-field profile of the magnet along the bore was measured along the bore with a hall effect sensor (Lakeshore Gaussmeter 455 DSP) and the sample positions were determined to place the sample center at 1 T, 0.1 T and 0.01 T. Field gradients at these positions amount to roughly 0.6 T cm^{-1}, 0.04 T cm^{-1} and 0.003 T cm^{-1}, respectively. The sample filling height was 3 cm. Small angle excitation spectra for ^{15}N-hyperpolarized samples were then measured at high field, and the samples were shuttled to the low field positions in-between scans. The residence time at high field τ_{HF} was 15s, whereas the residence time at low field τ_{LF} was 60s. Again, the correction procedure was applied to the signal integrals thus measured, prior to fitting:

For low field T_1 measurements, the measured signal integrals $I_0(n)$ were corrected for losses due to small flip angle pulsing and for signal loss during the high field residence periods, according to

$$I_{corrected,LF}(n) = \frac{I_0(n)}{\left(\cos\theta e^{-\frac{\tau_{HF}}{T_{1,HF}}}\right)^{(n-1)}} . \qquad (S8)$$

The T_1 values previously measured at the high field position ($T_{1,HF}$) are used during this correction. Further, a scaled time axis τ_{LF} is introduced, reflecting only the time portion spent at low field is used.

$$\tau_{LF}(n) = t(n) - \tau_{HF}(n-1) \qquad (S9)$$

The fit is then performed, according to

$$I_{corrected,LF}(n) = M_0 e^{-\frac{\tau_{LF}(n)}{T_{1,LF}}} , \qquad (S10)$$

to extract the low field T_1 value $T_{1,LF}$.

Again, a non-uniform weighing of the data points was used. For $I_{corrected,LF}(n)$, the error estimate

$$\Delta I_{corrected,LF}(n) = \frac{I_0(n=1)}{\left(S/N\right)_{n=1}} \frac{1}{\left(\cos\theta e^{-\frac{\tau_{HF}}{T_{1,HF}}}\right)^{(n-1)}} \quad (S11)$$

was used, and data point were weighed according to

$$w_{LF}(n) \propto \left(\Delta I_{corrected,LF}(n)\right)^{-2}. \quad (S12)$$

^{15}N-T_1 field dependence for compound 1a

For compound **1a**, the ^{15}N-T_1 was measured at four different fields, as described in the preceding section. The results already contained in Table 1 of the main article are reproduced in Figure S1, for facilitated inspection.

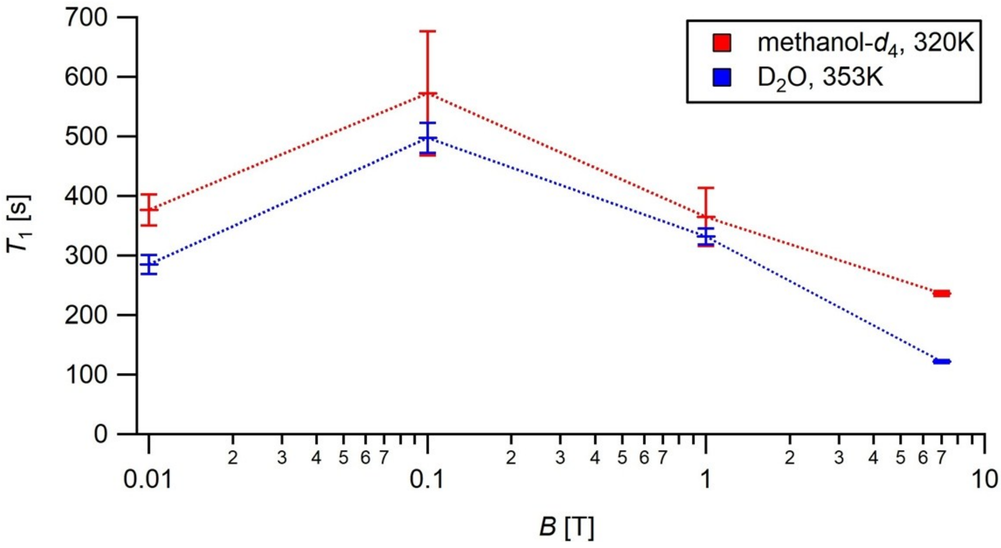

*Figure S1: Field dependence of the ^{15}N-T_1 for compound **1a**. Measurements were performed on freshly hyperpolarized samples without catalyst removal in two solvent/catalyst systems (as detailed in Table S1). Red: 4mM [Rh(dppb)(COD)][BF_4] in methanol-d$_4$, 320 K; blue: 0.5 mg/mL NAC@Rh in D_2O, 353 K. Dotted lines are added as a guide to the eye.*

Signal assignment and coupling values in the hydrogenation products 1a and 2a

Chemical shift ref.: CHD_2 (MeOD) = 3.31 ppm, or CD_3 (MeOD) = 3.31 ppm in ^{2}H spectra.

1a

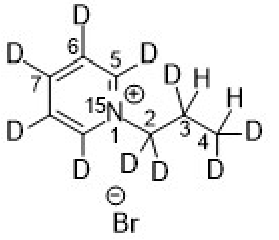

^{15}N NMR (D_2O, 353K, 30.4 *MHz*) = 213.2 ppm (d)

^{1}H NMR (D_2O, 353 K, 300.13 *MHz*) = 2.61 ppm (H3, dd), 1.51 ppm (H4, d)

$^3J_{NH}$ = 2.9 Hz, $^4J_{NH}$ < 0.3 Hz, $^3J_{HH}$ = 7.4 Hz

2a

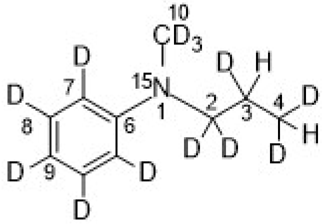

^{15}N NMR (MeOD+D_2O (1:1), 320K, 30.4 *MHz*) = 55.7 ppm (d)

^{1}H NMR (MeOD+D_2O (1:1), 320 K, 300.13 *MHz*) = 1.49 ppm (H3, dd), 0.84 ppm (H4, d)

$^3J_{NH}$ = 1.8 Hz, $^4J_{NH}$ < 0.2 Hz, $^3J_{HH}$ = 7.5 Hz

References

[1] P. J. Rayner, M. J. Burns, A. M. Olaru, P. Norcott, M. Fekete, G. G. Green, L. A. Highton, R. E. Mewis, S. B. Duckett, *Proc. Natl. Acad. Sci. U.S.A.* **2017**, *114*, E3188-E3194.

[2] H. Nonaka, M. Hirano, Y. Imakura, Y. Takakusagi, K. Ichikawa, S. Sando, *Sci. Rep.* **2017**, *7*, 40104.

[3] G. Compain, A. Martin-Mingot, G. Frapper, C. Bachmann, M.-P. Jouannetaud, S. Thibaudeau, *Chem. Commun.* **2012**, *48*, 5877-5879.

[4] R. A. Green, R. W. Adams, S. B. Duckett, R. E. Mewis, D. C. Williamson, G. G. R. Green, *Progress in Nuclear Magnetic Resonance Spectroscopy* **2012**, *67*, 1-48.

[5] aS. Korchak, S. Yang, S. Mamone, S. Glöggler, *ChemistryOpen* **2018**, *7*, 344-348; bS. Korchak, S. Mamone, S. Glöggler, *ChemistryOpen* **2018**, *7*, 672-676.
